# Utilization of household food waste for the production of ethanol at high dry material content

**DOI:** 10.1186/1754-6834-7-4

**Published:** 2014-01-08

**Authors:** Leonidas Matsakas, Dimitris Kekos, Maria Loizidou, Paul Christakopoulos

**Affiliations:** 1 Biotechnology Laboratory, School of Chemical Engineering, National Technical University of Athens, 5 Iroon Polytechniou Str, Zografou Campus, 15780 Athens, Greece; 2 Unit of Environmental Science and Technology, School of Chemical Engineering, National Technical University of Athens, 5, Iroon Polytechniou Str, Zografou Campus, 15780 Athens, Greece; 3 Department of Civil, Biochemical and Chemical Process Engineering, Division of Sustainable Process Engineering, Environmental and Natural Resources Engineering, Luleå University of Technology, SE 971 87 Luleå, Sweden

**Keywords:** Ethanol, Liquefaction, Saccharification, Household food waste, Residue solids, Subsequent fermentation, *Saccharomyces cerevisiae*

## Abstract

**Background:**

Environmental issues and shortage of fossil fuels have turned the public interest to the utilization of renewable, environmentally friendly fuels, such as ethanol. In order to minimize the competition between fuels and food production, researchers are focusing their efforts to the utilization of wastes and by-products as raw materials for the production of ethanol. household food wastes are being produced in great quantities in European Union and their handling can be a challenge. Moreover, their disposal can cause severe environmental issues (for example emission of greenhouse gasses). On the other hand, they contain significant amounts of sugars (both soluble and insoluble) and they can be used as raw material for the production of ethanol.

**Results:**

Household food wastes were utilized as raw material for the production of ethanol at high dry material consistencies. A distinct liquefaction/saccharification step has been included to the process, which rapidly reduced the viscosity of the high solid content substrate, resulting in better mixing of the fermenting microorganism. This step had a positive effect in both ethanol production and productivity, leading to a significant increase in both values, which was up to 40.81% and 4.46 fold, respectively. Remaining solids (residue) after fermentation at 45% w/v dry material (which contained also the unhydrolyzed fraction of cellulose), were subjected to a hydrothermal pretreatment in order to be utilized as raw material for a subsequent ethanol fermentation. This led to an increase of 13.16% in the ethanol production levels achieving a final ethanol yield of 107.58 g/kg dry material.

**Conclusions:**

In conclusion, the ability of utilizing household food waste for the production of ethanol at elevated dry material content has been demonstrated. A separate liquefaction/saccharification process can increase both ethanol production and productivity. Finally, subsequent fermentation of the remaining solids could lead to an increase of the overall ethanol production yield.

## Background

The environmental crisis and the shortage of fossil fuels have turned public attention to the utilization of other forms of energy, which are environmentally friendly and renewable, such as bio-ethanol [1,2]. During recent years research has focused on the so-called second-generation biofuels, where wastes or by-products are utilized as raw material, compared to the first-generation biofuels where sugars and starch were utilized. This way, increasing pubic concerns about utilizing food sources for the production of biofuels can be solved, as the utilization of either sugars or corn for the production of biofuels have contributed to the increase of their price worldwide, resulting in severe problems for the poorer countries. All these concerns led to a rapid increase in research to utilize low-cost by-products and wastes as raw material [3-6]. Lignocellulosic biomass represents great potential to be utilized as raw material due to the high amounts produced every year [7], and can be derived from woody or agricultural residues such as wheat straw, corn cobs, bagasse, rice straw, et cetera. The main challenge of utilizing lignocellulosic biomass is efficient sugar release, mainly from cellulose. In order to achieve this, an efficient pretreatment step followed by enzymatic hydrolysis have to be applied [8,9]. Generally, the pretreatment process contributes to increased costs of the whole process [10].

A different and alternative source of raw material for the production of biofuels could be the utilization of municipal organic wastes and especially household food wastes (HFW). Taking into account that the total quantity of HFW for the EU-27 during 2006 is estimated to be 37.7 Mt, which accounts for approximately 76 kg per capita and represents 42% of the total amount of food wastes generated in the EU [11], it is clear that they represent a challenge concerning their disposal, as well as an attractive raw material for the production of biofuels. Moreover, there is a trend of increasing the quantities of total food wastes produced (which are coming from both domestic, manufacture, food service/catering and retail/wholesale sectors), which, according to the European Commission (EC), will rise from 89.3 Mt in 2006 to 126.2 Mt by 2020 [11]. A common practice of HFW management is landfill disposal, which is causing severe environmental problems (such as greenhouse gas emissions) and shortage of disposal places [6,12,13]. Other practices are utilization as animal feed (which can raise hygiene issues) and soil conditioners-fertilizers (which can cause severe pollution to surface and underground water) [6,12,14]. Alternatively HFW can be used for the production of bio-based (green) chemicals and bio-energy (for example, biogas and ethanol) [15,16]. Until now, most of the research dedicated to HFW utilization was focused on biogas production.

Utilization of HFW as raw material represents a great challenge, as both the collection of generated HFW from multiple places [13] and post-collection treatment are difficult processes. Moisture and soluble sugars can make HFW an easy target for microorganisms, leading to their severe degradation. Another challenge is the heterogeneity that HFW present [13,14], which is highly affected by the source from which the wastes are derived. Nutritional habits and season of collection can also affect the composition of the HFW. Generally, fruits and vegetables represent a significant portion of the wastes [6,17]. Finally, one important issue to be solved is the proper education of the public in order to achieve low presence of contaminants (for example, plastics, metal et cetera) during source separation of HFW.

Concerning the utilization of HFW, there are some reports where different types of pretreatment, such as acid, alkali and thermal, have been used in order to increase cellulose digestibility [18-20]. Despite the fact that a pretreatment process can increase digestibility of cellulose, the soluble sugars can be degraded forming various inhibitors (such as furfural), especially if the pretreatment is performed at harsher conditions and in the presence of alkali.

The aim of this work was the utilization of source-separated HFW for the production of ethanol, at high dry material (DM) levels in order to achieve high ethanol production. Utilization of HFW at high DM levels results in a very viscous mash, where only solid-state cultivation can be applied, which presents many disadvantages including difficulties for process scaling-up and ethanol recovery [21]. In order to overcome this obstacle, an enzymatic liquefaction/saccharification process prior to fermentation, employing commercial cellulases solution (Celluclast®1.5 L and Novozym 188) was applied. During this process the viscosity of the high solid-content substrate was rapidly reduced, enabling submerged fermentation. No pretreatment prior to enzymatic saccharification was applied, in order to minimize the soluble sugar degradation. Finally, in order to maximize the ethanol yield a subsequent treatment and fermentation of the remaining solids (residue) was applied. During recent years, a new gravimetric mixing system has been successfully applied for the liquefaction of pretreated lignocellulosic feedstock at high DM content and was used in the present study [22,23].

## Results and discussion

### Fermentation of saccharified HFW

Table 1 shows the HFW composition obtained. HFW has potential to be utilized as raw material, as cellulose content is quite high and soluble sugars, such as glucose, fructose and sucrose are present and can be readily converted to ethanol. According to the literature, most researchers are analyzing food wastes by measuring the chemical oxygen demand (COD), biological oxygen demand (BOD), volatile solids (VS) et cetera [18-20,24-26], especially when they are utilized for biogas production. Though these values can provide important information about the raw material, for the ethanol production processes it is more important to know the proportion of soluble and insoluble sugars, as well as the type of insoluble polysaccharides, in order to apply the most appropriate enzymatic hydrolysis treatment.

**Table 1:** Composition of HFW

**Fraction**	**% w/w**
Soluble	33.81 ± 0.42
*Glucose*	4.39 ± 0.20
*Fructose*	3.47 ± 0.12
*Sucrose*	4.38 ± 0.10
*Total reducing sugars*	12.54 ± 0.93
*Protein*	0.54 ± 0.01
Fats	11.91 ± 0.68
Crude protein	10.51 ± 0.37
Pectin	3.92 ± 0.33
Cellulose	18.30 ± 0.19
Hemicellulose	7.55 ± 0.39
Klason lignin	2.16 ± 0.25
Ash	11.03 ± 0.42

Initial moisture content was 1.03 ± 0.20% w/w.

The values are given as mean ± SD.

Composition of food wastes can present a wide variety. Zhang and Richard [27] utilized a food-waste sample from a composting site of a University with a composition of 23.3% w/w total reducing sugars, 34.8% w/w starch and 1.6% w/w fibers and employed mainly amylases for its saccharification. Moon *et al.*[6] mentioned a high starch (30.1% w/w) and fiber content (14.9% w/w) with total reducing sugars of 17.6% w/w making it necessary to utilize both amylases and cellulases to treat it, whereas a high starch content (63.9% w/w) combined with low cellulose amounts was reported by Yan *et al.*[12] for the HFW sample that was used in their experiments.

**Figure 1:**
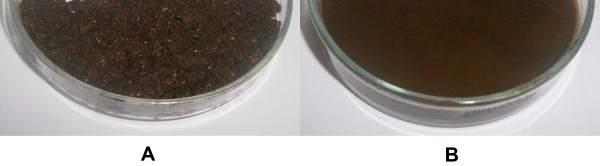
**Effect of enzymatic liquefaction/saccharification on household food wastes (HFW) at 45% dry material (DM). (A)** Prior to liquefaction/saccharification; **(B)** after 8 h of liquefaction/saccharification. Liquefaction of HFW was conducted for 8 h at an initial DM content of 45% w/v at 50°C. The enzyme load applied was 10 unit/g DM of a mixture of Celluclast® 1.5 L and Novozym 188 at a ratio of 5:1 v/v.

**Figure 2:**
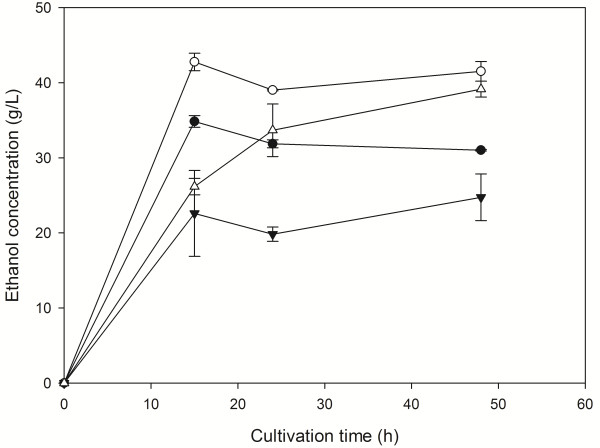
**Production of ethanol from liquefied household food waste (HFW).** Time course of ethanol production from HFW at 35% (solid circles) and 45% (open circles) initial dry material (DM) content with liquefaction/saccharification and at 35% (solid triangles) and 45% (open triangles) without liquefaction/saccharification.

The liquefaction/saccharification process was performed for 8 h at an initial DM content of 45% w/v followed by fermentation at two different initial DM contents (35% and 45% w/v). As can been evidenced from Figure 1, HFW was fully liquified after 8 hours of enzymatic treatment. This fact is also supported by the difference in the viscosity measured at an angular velocity of 10 rad/s before and after the enzymatic treatment, which decreased from 2790 Pa · s to 67.5 Pa · s, respectively. Maximum ethanol production in both DM contents was observed after 15 h of fermentation (Figure 2) and found to be 34.85 g/L (35% w/v DM) and 42.78 g/L (45% w/v DM) with a volumetric productivity of 2.32 g/L · h (35% w/v DM) and 2.85 g/L · h (45% w/v DM) (Table 2). Cellulose hydrolysis at the end of the fermentation reached 50.27% of the initial cellulose content in raw material. Considering this, the obtained yields (*Y*_*p/s*_) were 0.443 g/g and 0.423 g/g at 35% and 45% w/v DM respectively. The highest ethanol yield obtained when the fermentation was performed at 35% w/v DM could be attributed to the better mixing conditions.

**Table 2:** **Results of ethanol production during cultivation of *S. cerevisiae *** **on HFW**

**Initial dry material**	**Separate liquefaction step**	**Ethanol production**	**Ethanol productivity**	**% of the maximum theoretical yield**^**a**^	**% of the maximum theoretical yield**^**b**^
**(% w/v)**		**(g/L)**	**(g/L · h)**		
35	+	34.85 ± 0.55	2.32 ± 0.04	59.82 ± 0.94	159.21 ± 2.51
45	+	42.78 ± 0.83	2.85 ± 0.06	57.12 ± 1.10	151.91 ± 2.93
35	-	24.75 ± 2.20	0.52 ± 0.05	42.48 ± 3.78	113.07 ± 10.05
45	-	39.15 ± 0.75	0.82 ± 0.02	52.28 ± 1.00	139.07 ± 2.66

% Maximum theoretical ethanol yield, calculations were; ^a^based on the maximum ethanol that could be produced from the soluble and the cellulosic sugars; ^b^based on the maximum ethanol production from the soluble sugars only.

The values are given as mean ± SD.

With no use of the separate liquefaction step the ethanol production reduced by 28.98% and 8.49% when fermentation performed at 35% and 45% w/v initial DM, respectively. The use of the liquefaction/saccharification step was also associated with a significant increase in ethanol volumetric productivities (Table 2), mainly due to the partial cellulose hydrolysis which enabled reduction of viscosity and better mixing conditions of the fermenting raw material [28]. Same enhancement in ethanol production efficiency was also demonstrated by Kim *et al.*[25], who observed an increase in ethanol yield from 0.31 g/g to 0.43 g/g total solid when applying a Separate Hydrolysis and Fermentation (SHF) process instead of Simultaneous Saccharification and Fermantation (SSF) process on cafeteria food waste. Manzanares *et al.*[29] also found that with increasing initial DM content, a separate saccharification step improves fermentation of liquid hot water-pretreated olive-pruning biomass. Finally, Hoyer *et al.*[30] reported that with increasing DM content, even 4 h of saccharification could significantly improve fermentation of softwoods.

Ethanol production efficiency during this work was higher than that compared to Moon *et al.*[6] who performed a 3-h liquefaction process of food waste using both carbohydrases and amyloglucosidases where the ethanol production reached 29.1 g/L (Table 3). Walker *et al.*[31] utilized food wastes from starch-containing food and after saccharification with amylases the overall ethanol production was 8 g/L. Uncu and Cekmecelioglu [14] achieved 32.2 g/L ethanol production after 59 h of fermentation using food wastes treated for 6 h with amylases. Jeong *et al.*[32] reached 40.59 g/L ethanol production after 24 h of fermentation on food wastes hydrolyzed for 8 h with enzymes, using the fermentative microorganism *Saccharomyces coreanus*. When *Pichia stipitis* was added as a co-fermenting microorganism, ethanol production increased up to 48.63 g/L but the obtained productivities were lower than those of the present work. Cekmecelioglu and & Uncu [33] reported an ethanol production of 23.3 g/L after 48 h of cultivation on kitchen wastes saccharified for 6 h. Yan *et al.*[12] reported an ethanol production of 81.5 g/L from a saccharified high starch containing raw material (starch content was 63.9% w/w) using a high glucoamylase load (142.2 unit/g). Finally, Kim *et al.*[34] achieved 57.5 g/L ethanol production after 14 h of fermentation using starchy food waste saccharified for 4 h with amylases.

**Table 3:** Production of ethanol from food wastes from different sources

**Source of food waste**	**Carbohydrate content (% w/w)**		**Ethanol yield parameters**		**Reference**
	**Soluble**^**a**^	**Fiber**	**Concentration (g/L)**	**Productivity (g/L · h)**	
Cafeteria	n.a.	n.a.	n.a.	n.a.	[25]
Cafeteria	47.7	14.9	29.1	1.94	[6]
Dining center	n.a.	n.a.	8	n.a.	[31]
Cafeteria and houses	69		32.2	0.55	[14]
Cafeteria	n.a.	n.a.	48.63	2.03	[32]
Food courts	57.6		23.3	0.49	[33]
Dining room	63.9	1.98	81.5	1.36	[12]
Cafeteria	n.a.	n.a.	57.5	4.11	[34]
Houses	12.24	18.30	42.78	2.85	This work
Houses	12.24	18.30	34.85	2.32	This work

^a^Both soluble sugars and starch; n.a., not available.

### Pretreatment and fermentation of the solid residue

At the end of the fermentation there is a remaining solid fraction that was not converted to ethanol. This solid fraction contains unhydrolyzed cellulose which practically is lost from the ethanol production process. In order to increase the overall biofuel yield of the raw material’s mass, these solids could be further utilized. Some research has proposed the utilization of the remaining solids after fermentation and ethanol distillation for the production of biogas on kitchen waste [35], oat straw [36], wheat straw [37,38] and corn stover [39].

As it is described in the Methods section, after the end of the fermentation at 45% initial DM the solids were removed from the fermentation broth. This solid fraction contains the unhydrolyzed cellulose fraction, which could be further utilized for the production of ethanol in order to increase the overall production yield. The high degree of recalcitrance of this fraction [40] makes a pretreatment process prior to liquefaction/saccharifiacation necessary. During this study, hydrothermal pretreatment with the presence of acetic acid as a catalyst was applied [41,42]. After the pretreatment, solids were separated from the liquid fraction and washed with distilled water in order to remove the catalyst and other inhibitors formed during pretreatment. Inhibitor removal is necessary in order to decrease the stress to the fermenting microorganism, allowing higher fermentation rates and ethanol production efficiency [43-47].

As has been previously discussed cellulose hydrolysis reached 50.27% of the initial presented cellulose in HFW whereas the cellulose content of residue was 14.75% w/w. During the hydrothermal pretreatment process 42.73% of the initial mass of the residue was solubilized. Cellulose content of the pretreated residue was 16.31%, indicating a 36.68% of cellulose solubilization during the pretreatment.

**Figure 3:**
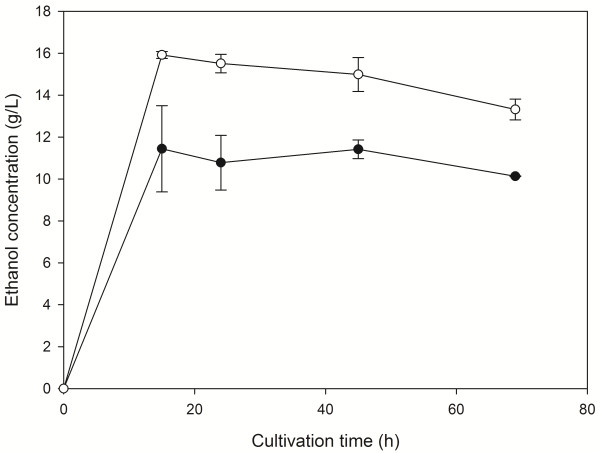
**Production of ethanol from liquefied residue.** Time course of ethanol production from hydrothermally pretreated residue at 35% (solid circles) and 45% (open circles) initial dry material content.

During fermentation of the pretreated residue at 35% and 45% w/v, maximum ethanol concentrations of 11.44 g/L and 15.92 g/L, respectively, were observed after 15 h (Figure 3). Moreover, increasing substrate concentrations of the fermented residue was associated with an increase in ethanol productivity (Table 4). From the initial cellulosic fraction of the pretreated residue, 42.67% was hydrolyzed. The obtained yields (Y_p/s_) reached 0.423 g/g and 0.458 g/g at 35% and 45% DM respectively, which were almost identical with the yields obtained during fermentation of HFW.

**Table 4:** **Results of ethanol production during cultivation of *S. cerevisiae *** **on pretreated residue**

**Initial DM**	**Ethanol**	**Productivity**	**% of maximum theoretical**
**(% w/v)**	**(g/L)**	**(g/L · h)**	
35	11.44 ± 1.45	0.76 ± 0.10	35.34 ± 4.49
45	15.92 ± 0.12	1.06 ± 0.01	38.23 ± 0.28

The values are given as mean ± SD.

### Overall ethanol yields

**Figure 4:**
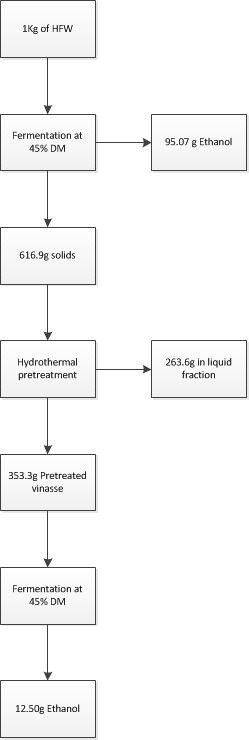
**Overall ethanol production yield.** Ethanol production yield after fermentation of liquefied household food wastes (HFW) at initial dry material (DM) content of 45% and subsequent fermentation of the residue. Prior to fermentation, residue was hydrothermally pretreated at 200°C for 10 minutes and liquefied.

Figure 4 presents the overall obtained ethanol yield after the fermentation of 1 kg of raw material using the two-stage sequential fermentation procedure (concerning the fermentations at 45% DM). In the first stage, 95.07 g of ethanol and 617.2 g of residue were obtained. The remaining residue was hydrothermally pretreated and the solid fraction was 353.5 g, whereas the other solids were dissolved to the liquid fraction. In the second stage after fermentation of the pretreated residue 12.51 g of ethanol could be obtained, thus the ethanol production yield could be increased from 95.07 g/kg DM to 107.58 g/kg DM, corresponding to an increase of 13.16%. The ethanol yield comparing to the maximum theoretical increased from 57.12% to 63.64%.

## Conclusions

In the current work the potential of utilizing source-separated HFW for the production of ethanol at high DM content was demonstrated. A liquefaction process prior to fermentation increased both ethanol production and ethanol volumetric productivity. Finally the subsequent fermentation of residue increased the overall ethanol production yield.

## Methods

### Raw material

HFW utilized as raw material during this work were source-separated from houses in Papagos-Cholargos Municipality, Athens, Greece. The wastes were dried *in situ* in a prototype house dryer designed and developed by the Unit of Environmental Science and Technology (UEST), School of Chemical Engineering, NTUA [48]. Dried HFW were milled with a small laboratory mill at an average particle size less than 3 mm. The composition of dried HFW is presented in Table 1.

### Reagents and enzyme solutions

All chemicals were of analytical grade. During this work a mixture of the commercial enzyme solutions from Novozymes A/S (Bagsværd, Denmark) Celluclast® 1.5 L (cellulases) and Novozym 188 (*β*-glucosidase) at a ratio of 5:1 v/v has been applied for the liquefaction/saccharification process. The activity of the mixture was measured according to the standard filter paper assay [49] and found to be 83 FPU/mL.

### Analytical methods

Total reducing sugars were measured by the dinitro-3,5-salicilic acid (DNS) method [50]. Monomeric sugars and ethanol were analyzed by HPLC (Shimadzu LC-20 AD, Kyoto, Japan) equipped with a refractive index detector (Shimadzu RID 10A, Kyoto, Japan). Monomeric sugar were analyzed utilizing an Aminex HPX-87P (300 × 7.8 mm, particle size 9 μm, Bio-Rad, Hercules, California) chromatography column, operating at 70°C with HPLC-water as a mobile phase at a flow rate of 0.6 mL/minute. Ethanol was determined by an Aminex HPX-87H (300 × 7.8 mm, particle size 9 μm, Bio-Rad, Hercules, California) chromatography column at 40°C, with a mobile phase of 5 mM sulfuric acid (H_2_SO_4_) at a flow rate of 0.6 mL/minute.

Soluble fraction was analyzed according to the official method of the National Renewable Energy Laboratory (NREL) [51]. The liquid fraction was further analyzed for sugars and proteins [52]. Moisture was analyzed according to Sluiter *et al.*[53], whereas crude fat, ash, protein and total starch content determination were conducted according to standard Association of Official Agricultural Chemists (AOAC) methods [54]. Pectin was determined according to Phatak *et al*. [55]. Finally, the cellulose, hemicellulose and (acid-insoluble) lignin content was determined according to Sluiter *et al.*[53]. It is worth mentioning that the HFW utilized during this work had no starch content. Analysis was carried out in triplicate. Apparent viscosity of the HFW before and after enzymatic treatment was determined by an Anton Paar Physica MCR rheometer apparatus (Anton Paar GmbH, Ashland, USA) as previously described [56].

### Hydrothermal pretreatment of residue

The remaining solids after fermentation of HFW were hydrothermally pretreated by microwave digestion equipment at 200°C for 10 minutes as previously described [41]. After the pretreatment, the solids were removed from the liquid fraction through vacuum filtration and washed in order to remove inhibitors formed during pretreatment. Finally, solids were dried at 60°C until constant weight reached.

### Enzymatic liquefaction and saccharification of HFW

Enzymatic liquefaction/saccharification of untreated HFW and hydrothermally pretreated residue was conducted in a liquefaction reactor which was designed and manufactured in-house. More specifically, the reactor consists of two vertical cylindrical chambers which are 6 cm wide and 25 cm diameter, with a rotating shaft driven by a 0.37-kW motor for the mixing of the material. The mixing shaft was programmed to shift from clockwise to anti-clockwise rotation every minute in order to achieve better mixing. Finally, the temperature was controlled by an external oil jacket.

The liquefaction/saccharification process was performed at initial DM concentration of 45% w/v for 8 h. The pH was adjusted to 5.0 by using 50 mM citrate-phosphate buffer and the enzyme load applied was 10 FPU/g DM. Finally, the temperature of the liquefaction/saccharification was set at 50°C. At the end of the liquefaction/saccharification process the whole slurry (also containing unhydrolyzed solids) was utilized for the fermentation experiments.

### Ethanol fermentation

Fermentations at 35% and 45% (w/v) DM of non-sterilized liquefied HFW or pretreated residue were performed in 100-mL Erlenmeyer flasks in an orbital shaker at 30°C with an agitation of 100 rpm. The fermenting microorganism was dry baker’s yeast (Yiotis, Athens, Greece), which was added at a concentration corresponding to 15 mg/g of initial DM. To evaluate the importance of the separate liquefaction/saccharification step, untreated HFW were fermented under the same conditions. Samples were taken at certain time intervals, centrifuged and analyzed for ethanol. All trials were carried out in duplicate.

When the HFW fermentation process was completed, the broth was filtrated under vacuum in order to remove the solids which were further washed with distilled water. The solids (residue) were dried at 60°C until constant weight reached and were further utilized for ethanol production after being hydrothermally pretreated (as previously described).

## Abbreviations

DM: Dry material; HFW: Household food wastes; HPLC: High performance liquid chromatography.

## Competing interests

The authors declare that they have no competing interests.

## Authors’ contributions

All authors (LM, DK, ML and PC) contributed jointly to all aspects of the work reported in the manuscript. All authors have read and approved the final manuscript.
